# “Look at Me, I Plan to Quit Smoking”: Bayesian Hierarchical Analysis of Adolescent Smokers’ Intention to Quit Smoking

**DOI:** 10.3390/healthcare8020076

**Published:** 2020-03-27

**Authors:** John N. Ng’ombe, N. Rodgers Nedson, Novan F. P. Tembo

**Affiliations:** 1 Department of Agricultural Economics and Rural Sociology, Auburn University, Auburn, AL 36830, USA; 2 Department of Agricultural Economics and Extension, University of Zambia, Lusaka 10101, Zambia; 3 School of Medicine, University of Zambia, Lusaka 10101, Zambia; nrodgersnedson@gmail.com; 4 Department of Public Health, University of Lusaka, Lusaka 10101, Zambia; darwibnovan2020@gmail.com

**Keywords:** tobacco smoking, smoking cessation, Hamiltonian Monte Carlo, Bayesian analysis, Zambia

## Abstract

The tobacco epidemic is one of the most prominent public health threats the world has ever faced. Public health policy that seeks to limit the problem may have to target not only the price of tobacco but also the initiation stage in a smoker’s life—the adolescent stage. Most research on teen smoking focuses on initiation and other stories. Moreover, what determines the desire to quit smoking among teens is not well understood, even though planning to quit smoking is an important stage toward successful cessation. This research contributes to healthcare literature by using Bayesian hierarchical techniques, estimated using Hamiltonian Monte Carlo (HMC) and its extension, the No-U-Turn Sampler (NUTS), to empirically identify what drives the intention to quit smoking among teen smokers in Zambia. Results suggest that, among the junior secondary school-going adolescent smokers in Zambia, about 63% have plans to quit smoking. We find socio-demographic characteristics and several tobacco-smoking-related factors as salient drivers of adolescent smokers’ plans to quit smoking. For policymaking, we recommend that school-going teen smokers should have access to smoking cessation aids to help them quit smoking. Most importantly, increased awareness of dangers of smoking, advice by health professionals, stringent public policies on smoking, as well as parental guidance could be useful to help adolescent smokers realize their quitting plans.

## 1. Introduction

While the tobacco use problem is more prevalent in developed countries, tobacco use is also rising in many developing countries [1,2]. For example, smoking rates are relatively low in sub-Saharan Africa (SSA), but projection rates indicate prevalence rates of around 39% by the year 2030 [3,4]. For Zambia, a Southern African country, tobacco use is on the rise in part due to lower cigarette pricing and tax rates than other countries in SSA [5]. According to the World Health Organization (WHO) [6], the most effective way to decrease cigarette consumption and its prevalence is to increase cigarette taxes. Additionally, Stoklosa et al. [5] suggest that increasing cigarette tax with corresponding price increases could reduce cigarette use in Zambia. However, Stoklosa et al. [5] assert that efforts to reduce cigarette use in Zambia are hampered by the availability of cheaper tobacco substitute brands. For example, about 39% of Zambian smokers smoke such brands as roll-your-own rather than factory-made cigarettes, and among them, about 88% say they do so because it is cheaper [3]. This means that, like most smokers, adolescents may not find it hard to access tobacco in Zambia and other developing countries in SSA. Based on the 2007 Global Youth Tobacco Survey (GYTS), 10.5% of Zambian students aged between 13 and 15 years were smokers [7].

In addition, although measures to increase taxes on cigarettes may be effective at reducing tobacco use, Zambia’s tax rate on cigarettes does not match WHO recommendations. The WHO endorses a tax share of 75% [6] while by 2016, Zambia’s tax share was, on average, about 37% of the retail price of cigarettes [8]. Thus, even if Zambia implemented WHO recommendations, tobacco consumers would still have the opportunity to access cheaper, locally cultivated tobacco [5], thereby undermining tobacco control policies. Given these social concerns, tobacco control policies may have to target not only the price of tobacco but also the behavioral origin of smoking in a smoker’s life—the adolescent stage—the stage in life at which smoking is initiated and where the addictive nature of the habit makes quitting particularly hard [9]. 

Cumulatively, there have been other attempts to reduce tobacco use in Zambia (e.g., the National Public Health Act of 1992, 2008 smoking ban at public places, 2009 smoking ban enforcement, increased cigarette taxes, and others [2]). Nonetheless, tobacco-related deaths in Zambia increased from 3000 per year in 1990 to 8000 per year in 2015 [10]. Projections indicate that children and young people alive today from developing countries are to bear the most burden of tobacco-related morbidity and mortality in the near future [8]. For example, the WHO forecasts an increased number of smokers in Zambia from 1.2 million in 2015 to 1.5 million in 2025, which suggests that tobacco use in Zambia is a public health concern largely expected to spill into the future. 

While scientific evidence indicates tobacco use is one of the leading causes of preventable death in the world, people still smoke, and even so, smoking is usually initiated during adolescence [9]. Shafey et al. [9] and Brook et al. [11] contend that, while smoking-related mortality mostly occurs during adulthood, smoking tobacco is usually initiated during adolescence, and the habit’s addictive nature makes quitting extremely difficult. By 2020, smoking-attributable deaths are expected to reach seven million a year and at least eight million each year by 2030 [9]. However, most people initiate their smoking behavior as adolescents or young adults. Given these concerns, important research questions moving forward are whether adolescent smokers have any plans to quit smoking and what factors explain their intentions to quit smoking. Following [12,13], the theory of planned behavior suggests that a person’s behavior is determined by the intentions to engage in that behavior as well as the perceived behavioral control over a habit. Additionally, [12] suggests that people’s behavioral intentions capture the motivational factors of a given behavior, and that intentions indicate how hard people are willing to try in addition to the amount of effort they plan to exert so as to perform the behavior. While planning to quit smoking is an important stage towards successful cessation, most research on adolescents’ smoking behavior has focused on initiation and other important issues (e.g., [7,11,14,15,16,17,18,19,20]. Motivated by the theory of planned behavior, prevalent smoking concerns, as well as a lacuna in empirical literature that explains adolescent smokers’ intentions to quit smoking, this study seeks to address the research questions stated a few moments ago. 

The objective of this paper is to elicit opinions about adolescent smokers’ plans to quit smoking. Specifically, we determine among adolescent smokers the proportion that intends to quit smoking as well as examine what drives their intentions to quit smoking in Zambia. Unless research reveals more information about what drives adolescent smokers’ plans to quit smoking, the WHO’s 2025 targets of 30% relative reduction in prevalence of tobacco use among persons aged at least 15 years [21] may not be achieved. In practice, knowledge about these factors may provide useful insights for tobacco prevention interventions and effective policy targeting that would help current teen smokers to successfully realize their quitting plans.

The main contributions of this research to the tobacco control and healthcare literature are both topical and methodological. First, despite tobacco smoking being the most preventable cause of death, there is a dearth of empirical research that explains youths’ intentions to quit smoking, even if smoking is mostly initiated during adolescence. We are the first to empirically and rigorously identify what drives the adolescents’ preferences to quit smoking in Zambia and other developing countries. This is important because, as governments try to achieve the WHO 2025 target of 30% relative reduction in prevalence of tobacco use among adolescents, understanding what drives adolescents’ intentions to quit smoking is necessary because it could help with effective targeting of more robust and proactive tobacco control policies. The second contribution is methodological, because the paper employs an empirical application of a Bayesian hierarchical logistic model estimated using Hamiltonian Monte Carlo—a modern Markov chain Monte Carlo (MCMC) procedure that uses the No-U-Turn Sampler (NUTS), which is a more efficient algorithm at estimating nonlinear Bayesian models [22,23]. A Bayesian hierarchical logistic regression is a class of regression models where the outcome is dichotomous and inference is under a Bayesian framework while taking advantage of information across groups of observations to reduce sensitivity of lower-level parameters to noise [22,24,25]. Our empirical application of HMC and NUTS is novel because these methods are state-of-the-art techniques and are more efficient than Gibbs samplers and variants of Metropolis–Hastings algorithms, especially because the Monte Carlo samples suffer less from between-chain autocorrelation [22,26]. Moreover, we use a Bayesian hierarchical framework instead of other approaches because it is exact in any sample size and allows individual intercepts to be centered. This reduces sensitivity of lower-level parameters to random noise, thereby assuring more credible results [27,28,29,30] 

## 2. Data

### Data Description

Data used in this study are from the most recent Global Youth Tobacco Survey (GYTS) [31] for Zambia, made available in 2018. The WHO-designed GYTS is a global standard survey that monitors youth tobacco use and tracks important tobacco control indicators. The GYTS is a cross-sectional survey of students that are in junior secondary schools. It is designed as a standardized tool to collect data on tobacco use among adolescents in countries around the globe. Thus, the GYTS is mainly a school-based survey of students commonly aged between 13 and 15 years [32].

The U.S. Centers of Disease Control and Prevention (CDC) and the WHO use a global standardized methodology when conducting the GYTS. Sampling included a two-stage cluster sample design with schools selected in Zambia with a probability that was proportional to enrollment size. Next, classes within the selected schools were chosen randomly. Eligible students for the survey were those that belonged to selected classes. Furthermore, only those that belonged to either a seventh, eighth, or ninth grade at the selected schools were finally surveyed. The overall response rate of all students surveyed was 55.7% [32].

The GYTS comprises 56 core questions, and the questionnaire covers such topics as young people’s knowledge and attitudes about smoking, prevalence of tobacco use, and access to cigarettes, among others. Numerous studies have used the GYTS for research because it offers uniform and nationally representative data (e.g., [33,34,35,36]). For Zambia, the GYTS had 3377 observations capturing students in grades seven, eight, and nine, of which 1964 were aged between 13 to 15 years. Because this study focuses on tobacco smokers, the respondents were those that indicated that they were smokers at the time of the survey. After omitting non-response observations, our final sample comprised of 640 students. 

A full description of the variables used in the analysis is presented in Table 1. We selected these variables based on a review of previous theory of planned behavior as well as tobacco use-related work. These include [7,12,13,14,15,16,17,18,19,20,37,38,39]. The response variable is intention to quit smoking, which equals 1 if the respondent wants/intends to quit smoking and 0 otherwise. The explanatory variables are presented below the response variable. 

We grouped explanatory variables into socio-demographic characteristics and tobacco smoking-related factors. Descriptive statistics of these variables are in Table 2. Table 2 indicates that students in the survey were from grades seven, eight, and nine. Students in grade seven contributed 39.8% of the total sample, while those from grade eight contributed 32.5%. The remainder (27.7%) were in grade nine. In this study, we assume our model’s intercepts vary by the variable grade because students from these three grades may not have similar characteristics, and thus their responses could be affected by grade-variation. Table 2 further shows that 63.3% reported they intended to quit smoking, while the rest (36.7%) did not. In terms of age, 4.8% of the students were aged 11 or below. About 58% of the respondents were aged at least 12 years up to 16 years old, and the rest were at least 17 years old. The GYTS is commonly conducted for students aged between 13 and 15 years [32]. However, our GYTS dataset had students younger than 13 years as well as those older than 17 years.

## 3. Materials and Methods

### 3.1. Conceptual Model

Following [40,41], in a logistic model, the underlying dependent variable $y_{ij}$ follows the Bernoulli distribution, y~Bin(1, π) assuming **x** as a vector of exogenous explanatory variables. The model is

(1) $$y_{ij}=\pi_{ij}+\varepsilon_{ij},$$

where *i =* 1, …, $N_{j}$ is the individual-level indicator, *j* = 1, *…, J* group of interest-level indicator, $\pi_{ij}$ is the probability of success for *i*th individual from *j*th group, and $\varepsilon_{ij}$ are individual level random errors. The probability of success is conditional on the influence of factors in **x**. Based on this information, the logit function is

(2) $$\mathrm{logit}\left(\pi_{ij}\right)=\log\left(\frac{\pi_{ij}}{1-\pi_{ij}}\right)=\alpha +x_{ij}^{{}^{\prime }}\beta$$

For Equation (2), $\varepsilon_{ij}{}^{\prime }s$ (as shown in Equation (1)) are assumed as independent with mean zero and $Var(\varepsilon_{ij})=\pi_{ij}\left(1-\pi_{ij}\right)$. Solving for $\pi_{ij}$ in Equation (2) yields

(3) $$\pi_{ij}\left(x;\beta \right)=\frac{e^{\alpha +x_{ij}^{{}^{\prime }}\beta }}{1+e^{\alpha +x_{ij}^{{}^{\prime }}\beta }}=\frac{1}{1+e^{-\left(\alpha +x_{ij}^{{}^{\prime }}\beta \right)}}$$

where the terms in Equation (3) are as defined previously. Partly because of its mathematical convenience, the logistic model is widely used in econometric applications [42]. Following [43], since logistic regression predicts probabilities instead of classes, it can be estimated using maximum likelihood. For a given set of data point x and observed class $y_{ij}$, the probability of the classification variable is either $\pi_{ij}\;$given y=1, or $1-\pi_{ij}\;$if y=0. Thus, the likelihood function of the logit model is

(4) $$f\left(y_{ij}|\alpha ,\;\beta ,\sigma_{\varepsilon }^{2}\;\right)=\prod_{i=1}^{n}\pi_{ij}{\left(x\right)}^{y}{\left(1-\pi_{ij}\left(x\right)\right)}^{\left(1-y\right)},\cdots y=0,\;1$$

The frequentist approach would require maximizing the log likelihood function in Equation (4) over the sample to obtain estimates for α and β. This paper adopts a Bayesian approach, which incorporates the hierarchical structure to better understand what drives adolescents’ preference to quit smoking. The Bayesian approach enables one to obtain model parameters’ posterior distributions. Following [22], posterior distributions allow one to make robust informative statements about their findings by using Bayesian credible intervals—the Bayesian equivalence of confidence intervals used in classical econometrics. Proponents of Bayesian inference posit that the frequentist approach draws its conclusions directly from the data. A frequentist approach interprets a confidence interval such as a 90% confidence interval as the range of values that would include 90% of parameter estimates if the data generating mechanism is repeated independently a copious number of times [29,30,44]. However, the Bayesian framework substitutes repeating experiments several times by advocating for a combination of prior information and data. It thus substitutes confidence intervals with Bayesian credible intervals, which are interpreted as the probability that a given estimate lies in the range of the values given in the data regardless of the data [44]. This presents a more intuitive interpretation of results regardless of how scarce the data are [27]. Motivated by these observations as well as earlier statements, this paper employs a Bayesian hierarchical logistic regression model. 

Our procedure is as follows. We consider the vector of parameters of interest as ϑ=[α, β]’, where α and β were defined previously. Using the likelihood function in Equation (4) and the prior distributions f(ϑ) for all parameters, we use Bayes’ rule to obtain the posterior density of all parameters using Equation (5)

(5) $$f\left(\vartheta |y_{ij}\right)\propto f\left(y_{ij}|\;\vartheta \right)f\left(\vartheta \right)$$

Equation (5) is based on regular Bayes’ rule, but in a hierarchical framework, as in our case, the prior density also depends on its lower level hyper-parameters, δ. The likelihood function also changes in a similar manner resulting in Equation (5) to become

(6) $$f\left(\delta ,\vartheta |y_{ij}\right)\propto f\left(y_{ij}|\;\vartheta ,\delta \right)f(\vartheta |\delta )f\left(\delta \right)$$

Obtaining the posterior distributions in Equation (6) by numerical integration methods, especially with multiple parameters, can be challenging, but the advent of MCMC methods makes estimation of Equation (6) easier. Further details about estimating Bayesian hierarchical models can be found in [22].

### 3.2. Empirical Model

Equation (2) is rewritten in a way to show the full empirical specifications. Our interest is to model the intention to quit smoking among adolescents to account for the between-grade heterogeneity in student responses. As discussed before, respondents in our data were from grades seven, eight, and nine; we specify our Bayesian hierarchical logistic regression model as one with varying intercepts according to grade. The full model is specified in Equations (7) and (8)

(7) $$\log\left(\frac{\pi_{ij}}{1-\pi_{ij}}\right)=\alpha_{0j}+\sum \beta_{k}x_{ik}$$

(8) $$\alpha_{0j}=\alpha_{0}+\mu_{j}$$

$$\;\;\beta_{k}\sim N\left(0,\;10\right)\;,\;\;\;\;\;\;\;\alpha_{0}\sim N\left(0,\;10\right),\;\;\;\;\;\;\;\;\;\;\;\sigma_{\mu }\sim Cauchy\left(0,\;2.5\right)\;$$

where $\alpha_{0j}$ is the varying intercept, $\beta_{k}$ indexes the *k*th coefficient of the *k*th explanatory variable, and $x_{ik}$ is the *k*th explanatory variable of the *i*th student in the sample ($x_{ik}$’s are explanatory variables shown in Table 2). The varying intercept model (Equation (8)) is the lower-level model showing that intercepts would vary according to $\alpha_{0}$ and the between-intercept error $\mu_{j}$, whose variance is $\sigma_{\mu }^{2}$. Following [22,40,45], we impose weakly-normal priors on $\alpha_{0}$ and $\beta_{k}$ because these can be either positive or negative, even if our null hypotheses for these are that they are zero. We further impose Cauchy priors for $\sigma_{\mu }^{2}$ (between-intercept variance represented by its standard deviation $\sigma_{\mu }$ shown in Equation (8)). The priors are chosen to provide little information, which conforms with [46] who posits that posterior standard deviations should be smaller than 10% of the corresponding prior standard deviations.

**Figure 1:**
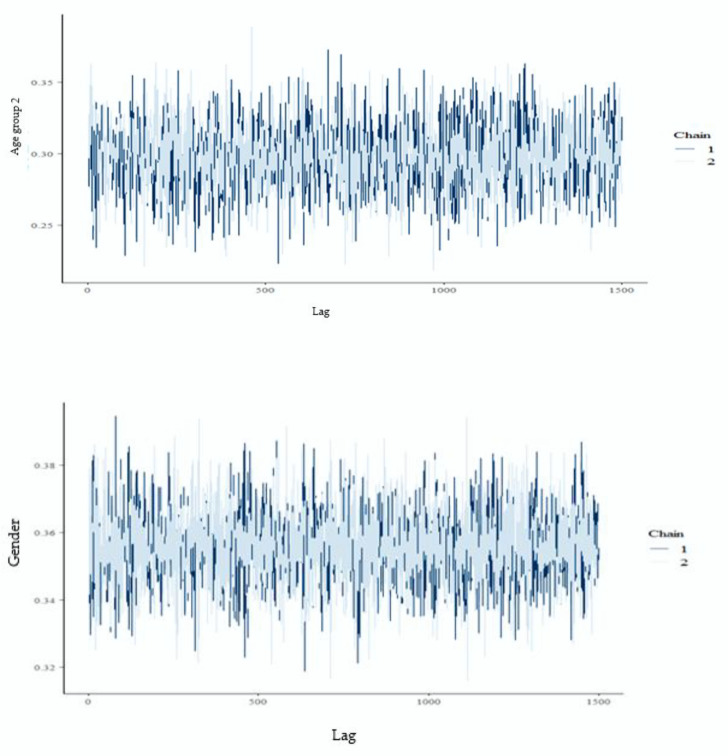
Trace plots for age group two and gender of the respondents. Chain 1 and chain 2 refer to the two Markov chains used in the MCMC techniques for all our estimations.

We then use HMC methods to sample from the posterior. As mentioned before, HMC uses NUTS, which is proposed to reduce the HMC dependency on its parameters and is more efficient than both the Gibbs sampler and the Metropolis–Hastings [23,47]. All estimations are conducted in R, RStudio, and Stan software [48,49,50]. Our HMC techniques involve two chains with a burn-in phase of 2500 to enable the Markov chains to forget their initial regions [27,29] with total iterations of 4000 per chain. We present convergence diagnostics to show that our MCMC chains converge to their target posterior distributions. Convergence diagnostics are shown in Figure 1. Since we have multiple independent variables, trace plots as convergence diagnostics for only two variables—gender and age group two—are shown in Figure 1. The trace plots for both variables indicate that MCMC chains exhibit good mixing, which suggests successful convergence. In Bayesian analysis, formal tests to check for convergence exist, and these include the Gelman–Rubin test [51], Geweke diagnostic [52], Raftery and Lewis [53], and many others. Thus, in addition to graphical methods, out of preference, we also apply the Gelman and Rubin test [51] to confirm convergence of the sequences. The Gelman–Rubin test checks whether parameter estimates are stationary, i.e., whether the Markov chains converged to their posterior distributions. The test compares the intra- and the inter-chain variation, and if the test statistic for all parameters is less than 1.10, then convergence is successful, otherwise, it is not [22,51,54]. The Gelman–Rubin test statistics are smaller than 1.004 for all parameters of the estimated models, which provides strong evidence of convergence—a result consistent with trace plots in Figure 1.

## 4. Results and Discussion

Estimation results are presented in Table 3. Column 1 presents variable names, while columns 2 and 3 respectively present mean odds ratios and their standard deviations. The Bayesian 95% credible interval is in column 4, while percentage effects are presented in column 5. Percentage effects display the percentage change in the odds of a respondent’s intention to quit smoking. 

The upper and the lower bounds of the Bayesian 95% credible intervals shown here are the 0.025% and the 0.975 quantiles of the corresponding posterior odds ratios derived as exponentiated posterior means. Though we report odds ratios in Table 3, the actual posterior means are shown in Table A1 in the appendix to conserve space. The reference value when interpreting the odds ratios in Table 3 is the value of one. An odds ratio greater than one implies an increase in the likelihood of one’s intention to quit smoking, while a value less than one means a decrease in the probability of one’s intention to quit smoking. In terms of statistical significance, we consider the odds ratios as statistically significant if their Bayesian credible intervals do not include the value of one, which is equivalent to parameter estimates (i.e., posterior means) being statistically significant if the credible intervals do not span zero. The varying intercepts in the model account for inter-grade variation of the multiple responses from students in each grade. This accounts for heterogeneity from the fact that students from some grades would intend to quit smoking more than average, while others would intend to quit smoking less than average. Posterior means of group-level effects are shown in the last row of Table A1.

Regarding socio-demographic characteristics, male students would more likely intend to quit smoking than female students. On average, male students’ odds of planning to quit smoking are about 42% more than female students, keeping other factors constant. This finding is consistent with [18], who found that female smokers exhibit more difficulty at sustaining long-term abstinence from smoking than males, which could be due to multiple-level bio-psycho-social factors. Smith et al. [18] contend that such factors could lead to psychiatric distress, which more likely stimulates some female smokers to smoke again, even when they attempt to stop smoking. We also find that students that are aged between 12 and 16 are, on average, more likely to plan to quit smoking than those aged 11 or below. Similar results are found for students aged at least 17 years. The odds of the intention to quit smoking are about 33% higher for students aged at least 17 years than those aged 11 or less, and this value ranges between 26.9% and 39.5% with probability of 95%, other factors held constant. These findings could be because older students are more likely to know the dangers of smoking than younger ones (under 11 years old) that may be still undergoing crucial periods of growth and development—thereby being more vulnerable and less likely to plan to quit. Our findings are in part consistent with [55,56,57]. Derby et al. [55], Hymowtz et al. [56], and Lee and Kahende [57] found a positive relationship between the likelihood to cease smoking and age.

For the tobacco smoking-related factors, we find that smoking an additional cigarette per day increases the odds of the intention to quit smoking by 5%, the value that ranges from 4.2% to 5.9% with a probability of 0.95, ceteris paribus. This could be in response to the negative mood smokers feel after smoking the last cigarette. Folan et al. [58] contend that smokers feel a negative mood change after smoking because they no longer get the effect on the brain to enable them function well. Folan et al. [58] further suggest smokers usually get relief from such unpleasant feelings once they smoke again, which makes them smoke again despite having planned to quit smoking. We find that students that smoked smokeless tobacco in last 30 days have higher odds (they range between 1.980 and 2.092 with probability of 0.95) of planning to quit smoking than otherwise, ceteris paribus. The more likely reason for this could be smokeless tobacco-associated lesions. Smokeless tobacco is usually associated with cavity lesions in smokers [59,60], which would perhaps result in students that smoke disliking these effects. Furthermore, students that have been smoking in the previous five years are found to be more likely to plan to quit smoking than those that have not, which suggests they could have been ready to quit in the past five years but, possibly because of such factors as nicotine addiction, they still smoke. As argued by Forlan et al. [58], smokers are susceptible to moderate or severe nicotine addiction and may have difficulty stopping tobacco use without serious assistance, which could be the case for students that have been smoking the past five years but strongly plan to quit.

In terms of students’ perception about the difficulty to quit smoking, results show that the odds of planning to quit smoking among respondents that believe it is difficult to quit smoking are higher than those that believe otherwise. Perhaps, these are such students that could have attempted to quit smoking but failed. As in Zhou et al. [61], quitting smoking is dynamic and involves a sequence of unsuccessful attempts before long-term abstinence. Our finding implies that there are students that believe quitting is difficult, something that requires serious cessation aids, such as serious media awareness about the dangers of smoking and other evidence-based methods. For example, we find that belief among students that smoking is harmful to health increases the odds of their plans to quit smoking by about 21%, a value that lies between 18.2% and 24.1% with a probability of 0.95, everything else held constant. This finding is plausible, as tobacco smoking is known to cause death and cancer-related mortality, even among young people [6,62], and thus knowledge of the dangers that smoking has on their health may lead students to have second thoughts about smoking – making them more likely to plan to quit. 

Results show that belief among students that smoking results in weight loss or weight gain since its initiation increases the odds of planning to quit smoking tobacco among school-going adolescents. These findings imply the need for school-goers that smoke to be kept well informed about weight-related health benefits associated with smoking cessation. Previous research indicates weight-related benefits that come from smoke cessation. While [63] indicate that nicotine is linked to increasing energy expenditure and decreased appetite, which is conducive to weight loss in smokers, [63] further posit that sometimes smoking increases insulin resistance, resulting in central fat accumulation and ultimately leading to weight gain in heavy smokers. Moreover, studies by [64,65,66] indicate that smokers gain weight following cessation because nicotine is an appetite suppressant of fat. However, overall benefits associated with smoking cessation, such as stronger and healthier heart and lungs, whiter teeth, younger skin, and better breath [67], would perhaps guarantee a better and lifelong future for teens despite possible post-cessation weight gain. Additionally, adolescent smokers may get around weight gain associated with smoke cessation by involving themselves in physical activities, buying healthy groceries, and controlling what they eat [67]. 

Students that believe school-going adolescent smokers should seek permission to smoke tobacco are less likely to plan to quit smoking than otherwise. Specifically, belief among students that school-going adolescent smokers should seek permission from adults to smoke decreases the odds of plans to quit smoking by 22%, and this value ranges between 18.2% and 24.1% when other factors are held constant. Moreover, those that believe smoking is safe for the first one or two years have higher odds of planning to quit smoking than otherwise. These results reveal how students underestimate the dangers of smoking, even though a day’s cigarette can be harmful to their health [68], which suggests the need to help them understand the dangers of smoking. We also find that the number of times students have smoked in-house or inside public buildings affects the probability of their intention to quit smoking. The more they smoke in-house or at public places, the higher are the odds of intending to quit smoking. This could be due to smoke-free warnings at public places or in enclosed buildings. Smoke-free laws and policies may reduce smoking prevalence among workers and smoking initiation among adolescent smokers [69]. Those that favor bans of smoking at public places are more likely to plan to quit smoking. Keeping other factors constant, there is a 95% probability that favoring a ban of smoking at public places increases the odds of one’s plans to quit smoking by about 163% on average, and this value ranges between 156.3% and 170.5%. Because tobacco-free laws motivate and help tobacco users to quit smoking and also thwart initiation of tobacco use among youths [70], students who support a ban of smoking at public places may indeed be more likely to have plans to quit smoking. 

Results further show that advice to stop smoking strongly increases the odds of adolescents’ plans to quit smoking. Receiving advice to stop smoking on average increases the odds of students’ plans to quit smoking by 188.4%, a result that varies between 180.2% and 196.6% with 95% probability, keeping other factors constant. While advice could be helpful, the smoker also needs to be motivated to do so, because quitting may be affected by other exogenous factors around the smoker’s habitat. For example, results further show that students that own cigarette brands or logos on cloth, attend cigarette advertisement events, or have in the past been offered free cigarettes before are more likely to plan to quit smoking than otherwise. Even students that have received lessons about dangers of smoking in the past are more likely to plan to quit smoking than otherwise. Our results reveal that teaching students about the dangers of smoking increases their odds of intending to quit smoking by 44.4%, a result that varies between 41% and 47.9% with a probability of 0.95, everything else held constant. These results highlight the role played by the social environment and education at influencing adolescents’ intentions to quit smoking. 

The number of cigarettes a student has smoked in life also has meaningful effects on their intention to quit smoking. We find that the number of cigarettes a student has smoked in life significantly affects his/her plans to quit smoking. Students that have smoked more cigarettes in life are, on average, less likely to quit smoking, which could be due to nicotine addiction. This finding is consistent with [71] who find that smokers are more likely to attempt to quit smoking if they have smoked fewer cigarettes in their lives. When cigarettes are sold near students’ residences or homes, the odds of students’ intention to quit smoking, on average, decrease by about 32%, a value that ranges between 30.4% and 33.7% with 95% probability. While cigarettes sold in close-proximity would be expected to boost their access, the price at which they are sold could be the main deterrence, especially for school-going adolescents. As in [72], a general environment of higher cigarette prices encourages quit attempts because it may limit access. 

Those that consider that youth groups are discouraging smoking are more likely to plan to quit smoking than otherwise. This suggests that youth groups such as School Unions or Associations may be strongly discouraging smoking among students, leading to students having plans to quit smoking. However, results suggest students that believe with conviction that religious groups discourage smoking are less likely to plan to quit smoking. This could be due to the belief that religious groups discouraging smoking may not be necessary for planning to quit smoking but rather getting involved in activities of such religious groups or institutions could help. For example, [73] find that teens that attend religious services have lower cigarette smoking rates than otherwise. Furthermore, we find that, when health workers explain the dangers of smoking to school-going smokers, it increases the latter’s odds of planning to quit smoking by about 59%, a result that ranges between 55.3% and 62.9%, with 95% probability, ceteris paribus. This highlights the important role health practitioners could play in helping to reduce smoking rates among students in schools. 

Most tobacco users desire to quit smoking. Some succeed to quit while others do not. The desire to quit smoking varies from person to person. Here, we find that students do attempt to quit smoking for several reasons that include: to improve health, to save money, or because their families dislike smoking tobacco. Results suggest quitting attempts to improve health, to save money, or because their families dislike smoking increase the odds of the students’ intention to quit smoking, on average, by about 166%, 81%, and 101%, respectively, when holding other factors constant. These results suggest increasing sensitization among youths about health hazards from smoking, spending their money on alternative items such as school needs, or encouraging families to discourage their children from smoking could perhaps contribute to control of tobacco use among adolescents. 

In terms of peer-effects, we find that having close friends that smoke decreases the odds of intending to quit smoking by 16.1%, a result that ranges from 14.8% and 18% with a probability of 0.95. Compared to having two parents that do not smoke, our results suggest that, when it is only a father who is a smoker, on average, it increases the odds of a student’s plans to quit smoking by about 32%, a result that varies between 28.6% and 36.3% with 95% probability. In contrast, when it is only a mother who smokes, the odds of planning to quit smoking among students decrease 65.2% more than when both parents do not smoke, a result that ranges from 63.2% and 67.1% with a 95% chance, ceteris paribus. These findings are plausible and illustrate how significantly the effects from peers and parents could foster or undermine students’ plans to quit smoking.

We also consider common brands of cigarettes that students smoke. These include Peter Stuyvesant, Roth-man, Consulate, Safari, roll-your-own, and one without any known brands. Compared to brandless cigarettes, smoking Peter Stuyvesant cigarettes reduces the odds of the intention to quit smoking by about 57%, and this value ranges between 55% and 59.1% with a probability of 0.95, other factors held constant. This could be due to higher prices of Peter Stuyvesant cigarettes than brandless cigarettes, because the former is factory-made and therefore more likely to be costlier and less affordable than the latter. In contrast, students that smoke Roth-man or Safari brands are more likely to plan to quit smoking than those that smoke brandless cigarettes. Additionally, smoking roll-your-own significantly increases odds of students’ plans to quit smoking relative to smoking brandless cigarettes. The reason for differences in the plans to quit smoking across different brands could be pricing of these products as well as the students’ perceptions about their health hazards. For example, [5] contend that local farmers in Zambia also produce and supply tobacco as part of the roll-your-own brand, a lower priced burnable tobacco product that is used to substitute highly priced factory-made tobacco. However, the ingredients of such products may not be scientifically known among smokers because such tobacco is locally produced [5] and may be consumed just after harvesting from farm fields, which could pose health risks. 

## 5. Conclusions

While the tobacco use problem is more prevalent in developed countries, smoking rates are rising in most developing countries, and projection rates indicate high pervasiveness rates of around 39% by the year 2030 [3,4]. Health policy that seeks to limit the problem may have to target not only the price of tobacco but also the common stage at which smoking is initiated—the adolescent stage. This research contributes to the health economics literature by using Bayesian hierarchical techniques to empirically identify what drives the intentions to quit smoking among adolescent smokers. 

Based on our findings, the following conclusions are drawn. First, the average number of school-going teen smokers that plan to quit smoking in Zambia is about 63%. This clearly reveals that a large number of adolescent smokers in Zambia plan to quit smoking, and to help them achieve their plans, smoking cessation aids need to be available for them. Such aids could include smoking cessation programs at schools, small enticements to people that successfully quit smoking, short sessions on dangers of smoking [74], and possible school visits by health professions to make students more aware about deleterious effects of tobacco use on their health. Second, socio-demographics characteristics shape students’ plans to quit smoking. For example, the finding that students aged 11 or less are less likely plan to quit smoking than those older than 11 suggests such young students require special care and assistance to quit smoking for a future active and healthy population. That female smokers are less likely to plan to quit smoking than their counterparts, is an important result. Consistent with [4,18], this finding implies the need for special attention on young female smokers through strengthening public health interventions, focusing on them to help them withstand the stress or the so-called bio-physical and social factors that may undermine their plans to quit smoking. 

Third, several tobacco smoking-related factors are found to strongly affect adolescents’ plans to quit smoking. Positive significant effects of the belief among students that smoking is harmful to health, or that smoking leads to weight gain/loss on students’ plans to quit smoking, suggest increased public awareness of the dangers of smoking in schools could on average motivate students to plan to quit smoking. On average, teaching students about the dangers of smoking or school visits by health workers to advise students to abstain from smoking would help encourage students to plan to quit smoking. The media are also possible avenues to do so. Obviously, the next step would be ensuring that they attempt to quit smoking, and how long they maintain abstinence is an important challenge for future research. 

While the strategies to help students plan to quit smoking proposed in this article could be effective at improving adolescents to quit smoking, peer effects and parental smoking behavior may undermine them. Most importantly, students whose closest friends smoke or those with only a mother who smokes were found less likely to plan to quit smoking. This result is consistent with [16,75,76]. Thus, efforts aimed at encouraging youths to quit smoking should also be directed at parents because they shape their children’s behavior. Additionally, efforts directed at peer influence such as discouraging students from involving or interacting themselves with smokers should also be encouraged. This would not only prevent children from quitting tobacco use but would also prevent them from being exposed to passive smoking, which is also dangerous to health. 

Based on our findings, an important implication for tobacco control policy is how students should be encouraged to quit smoking successfully. As discussed before, a possibility is introduction of smoking cessation programs at schools by governments or responsible agencies. Students should be incentivized to participate in such programs to help or aid their planned behavior toward actual quitting. They would have to be taught and raised under stricter public policies of smoking regulation [19] to aid their plans to quit smoking and, most importantly, to attempt to quit. Obviously, any student that plans to quit smoking will be more enthusiastic to follow quitting routines presented to them during cessation programs and more likely achieve their new planned behavior. This article does not scorn the need for higher taxes that other studies acclaim (e.g., [5] and others) but rather provides the non-price alternatives that complement them. We recommend increased sensitization against smoking among teens, including smoking locally farmed and produced tobacco products whose nicotine contents are unknown to the consumers, as a part of targeting effective tobacco control policies in developing countries. 

Future research should use richer datasets, such as longitudinal data, which would ideally add more evidence to adolescents’ smoking behavior, quit attempts, and their abstinence success rates. Our data are from grade seven, eight, and nine students. Perhaps, data that would include more school levels would be richer. However, our data are from a global survey designed as a standard tool by the CDCs, which makes it a robust dataset. In addition, the Bayesian hierarchical methods used in this study help to account for sources of uncertainty from both data and parameters. While the analysis was conducted for Zambia, our results provide general implications for readers and policymakers across the world at a time when the tobacco epidemic is one of the biggest public health threats the world has ever faced [21]. 

## Figures and Tables

**Table 1:** Definition of variables used in the analysis.

Variable Name	Variable Description
**Dependent variable**	
Intention/plan to quit smoking	Respondent intends or plans to quit smoking, equals 1 if yes, 0 o/w
**Explanatory variables**	
Socio-demographic characteristics	
Gender	Gender of respondent, equals 1 if male, 0 otherwise
Age group one	Respondent is aged 11 years or less
Age group two	Respondent is aged between 12 and 17 years old
Age group three	Respondent is aged at least 17 years old
Grade 7	Respondent is in grade 7
Grade 8	Respondent is in grade 8
Grade 9	Respondent is in grade 9
Tobacco smoking-related factors	
Cigarettes per day	Number of cigarettes smoked per day
Smokeless tobacco in past 30 days	Respondent smoked smokeless tobacco in past 30 days
Smoking for past 5 years	Respondent has been smoking for past 5 years
Difficult to quit smoking	Respondent believes it is difficult to quit smoking
Smoking is harmful to health	Respondent believes smoking is harmful to health
Gained weight following smoking	Respondent believes he/she has gained weight after smoking
Lost weight following smoking	Respondent believes he/she has lost weight after smoking
Smokers should ask permission to smoke	Respondent believes smokers should ask permission to smoke
Smoking is safe for 1 or 2 years	Respondent believes smoking is safe for one or two years
Number of times smoked in-house	Number of times respondent smoked inside a house
Number of times smoked inside enclosed public places	Number of times respondent smoked inside enclosed public places
Times smoked at outdoor public places	Number of times respondent has smoked at outdoor public places
Favors ban of smoking at public places	Respondent favors a ban of smoking at public places
Advised to stop smoking	Respondent received advice/help to stop smoking in the past
Owns cigarette brand logo	Respondent owns cigarette product brand logo
Attends cigarette ad events	Respondent attends cigarette advertisement events
Offered free cigarettes	Respondent is offered free cigarette
Taught dangers of smoking before	Respondent has before been taught about dangers of smoking
Number of cigarettes in life	The maximum number cigarettes the respondent has smoked in life
Cigarette sold near home	Respondent knows place(s) that sell single cigarettes near home
Youth groups discourage smoking	Youth groups are considered to be discouraging smoking
Health workers explained smoking dangers	Health worker explained dangers of smoking
Religious groups discourage smoking	Religious groups are considered to be discouraging smoking
Have not tried to stop smoking	Respondent has not tried to stop smoking
Tried to stop to improve health	Respondent tried to stop to improve own health
Tried to stop to save money	Respondent tried to stop to save money
Tobacco smoking-related factors	
Tried to stop because family dislikes it	Respondent tried to stop smoking because the family dislikes it
Closest friends smoke tobacco	Respondent’s closest friends smoke
Both parents smoke	Respondent’s father and mother smoke tobacco
Only father smokes	Respondent’s father smokes tobacco
Only mother smokes	Respondent’s mother smokes tobacco
No usual brand	Respondent uses no usual brand
Peter Stuyvesant	Respondent uses no usual brand uses Peter Stuyvesant brand
Roth man	Respondent uses Roth man’s brand
Consulate	Respondent uses consulate brand
Safari	Respondent uses safari brands
From own burns	Respondent smokes tobacco from burns

**Table 2:** Descriptive statistics of variables used in the study.

Variable Name	Mean	Std. Dev.
**Dependent variable**		
Intention to quit smoking	0.633	0.482
**Explanatory variables**		
Socio-demographic characteristics		
Gender	0.492	0.500
Age group one	0.048	0.215
Age group two	0.577	0.494
Age group three	0.375	0.485
Grade 7	0.398	0.490
Grade 8	0.325	0.469
Grade 9	0.277	0.448
Tobacco smoking-related factors		
Cigarettes per day	1.792	1.571
Smokeless tobacco in past 30 days	0.245	0.431
Smoking for past 5 years	0.230	0.421
Difficult to quit smoking	0.528	0.500
Smoking is harmful to health	0.441	0.497
Gained weight following smoking	0.209	0.407
Lost weight following smoking	0.444	0.497
Smokers should ask permission to smoke	0.358	0.480
Smoking is safe for 1 or 2 years	0.291	0.454
Number of times smoked in-house	2.261	1.595
Number of times smoked inside enclosed public places	2.319	1.486
Times smoked at outdoor public places	2.248	1.522
Favors ban of smoking at public places	0.359	0.480
Advised to stop smoking	0.809	0.393
Owns cigarette brand logo	0.313	0.464
Attends cigarette ad events	0.530	0.500
Offered free cigarettes	0.267	0.443
Taught dangers of smoking before	0.436	0.496
Number of cigarettes in life	2.289	2.004
Cigarette sold near home	0.405	0.491
Youth groups discourage smoking	0.356	0.479
Health workers explained smoking dangers	0.452	0.498
Religious groups discourage smoking	0.413	0.493
Have not tried to stop smoking	0.111	0.314
Tried to stop to improve health	0.227	0.419
Tried to stop to save money	0.059	0.237
Tried to stop because family dislikes it	0.209	0.407
Tobacco smoking-related factors		
Closest friends smoke tobacco	0.603	0.490
Both parents smoke	0.069	0.253
Only father smokes	0.213	0.409
Only mother smokes	0.047	0.212
Neither of the parents smokes	0.672	0.470
No usual brand	0.230	0.421
Peter Stuyvesant	0.077	0.266
Roth man	0.045	0.208
Consulate	0.059	0.237
Safari	0.006	0.079
From own burns	0.034	0.182
Number of observations	640	

**Table 3:** Odds ratios, standard deviations, Bayesian 95% credible intervals, and percentage effects.

Variable Name	Odds Ratios	Std. Dev.	95% Credible Interval		Percentage Effects (%)
Socio-demographic characteristics					
Gender	1.428	0.017	1.396	1.460	42.761
Age group two	1.347	0.032	1.286	1.410	34.737
Age group three	1.333	0.032	1.269	1.395	33.251
Tobacco smoking-related factors					
Cigarettes per day	1.050	0.004	1.041	1.059	5.005
Smokeless tobacco in past 30 days	2.035	0.029	1.980	2.092	103.463
Smoking for past 5 years	1.387	0.020	1.348	1.426	38.675
Difficult to quit smoking	2.262	0.026	2.213	2.311	126.158
Smoking is harmful to health	1.211	0.015	1.182	1.241	21.117
Gained weight following smoking	1.890	0.030	1.833	1.947	89.037
Lost weight following smoking	2.371	0.032	2.308	2.434	137.062
Smokers should ask permission to smoke	0.779	0.010	0.758	0.798	-22.141
Smoking is safe for 1 or 2 years	1.294	0.017	1.259	1.328	29.371
Times smoked in-house	1.125	0.005	1.115	1.135	12.482
Times smoked inside enclosed public places	1.163	0.006	1.152	1.173	16.256
Times smoked at outdoor public places	0.994	0.004	0.985	1.002	−0.647
Favors ban of smoking at public places	2.633	0.037	2.563	2.705	163.269
Advised to stop smoking	2.884	0.042	2.802	2.966	188.394
Owns cigarette brand logo	1.471	0.019	1.434	1.507	47.113
Attends cigarette ad events	1.889	0.021	1.848	1.932	88.933
Offered free cigarettes	1.031	0.015	1.004	1.060	3.135
Taught dangers of smoking before	1.444	0.017	1.410	1.479	44.371
Number of cigarettes in life	0.901	0.003	0.895	0.906	−9.932
Cigarettes sold near home	0.679	0.008	0.663	0.696	−32.062
Youth groups discourage smoking	1.209	0.016	1.177	1.240	20.905
Health workers explained smoking dangers	1.591	0.019	1.553	1.629	59.118
Religious groups discourage smoking	0.906	0.011	0.884	0.928	−9.373
Have not tried to stop smoking	0.733	0.013	0.708	0.759	−26.713
Tried to stop to improve health	2.661	0.042	2.579	2.745	166.061
Tried to stop to save money	1.812	0.045	1.726	1.901	81.197
Tried to stop because family dislikes it	2.013	0.031	1.952	2.075	101.284
Closest friends smoke tobacco	0.839	0.010	0.820	0.858	−16.116
Both parents smoke	0.984	0.021	0.943	1.028	−1.551
Only father smokes	1.324	0.019	1.286	1.363	32.426
Only mother smokes	0.348	0.010	0.329	0.368	−65.181
Peter Stuyvesant	0.429	0.010	0.409	0.450	−57.079
Roth man	1.407	0.041	1.327	1.489	40.737
Consulate	1.001	0.025	0.952	1.051	0.0738
Safari	1.381	0.096	1.201	1.569	38.053
From own burns	3.486	0.125	3.248	3.734	248.557

## Appendix A

**Table A1:** Posterior means, standard deviations, and Bayesian 95% credible intervals.

Variable Name	Posterior Mean	Standard Deviation	95% Credible Interval	
Socio-demographic characteristics				
Intercept	−3.720	0.670	−4.775	−2.672
Gender	0.356	0.012	0.333	0.379
Age group two	0.298	0.024	0.251	0.344
Age group three	0.287	0.024	0.239	0.333
Tobacco smoking-related factors				
Cigarettes per day	0.049	0.004	0.040	0.057
Smokeless tobacco in past 30 days	0.710	0.014	0.683	0.738
Smoking for past 5 years	0.327	0.015	0.298	0.355
Difficult to quit smoking	0.816	0.011	0.794	0.838
Smoking is harmful to health	0.192	0.012	0.167	0.216
Gained weight following smoking	0.637	0.016	0.606	0.666
Lost weight following smoking	0.863	0.014	0.836	0.890
Smokers should ask permission to smoke	−0.250	0.013	−0.277	−0.226
Smoking is safe for 1 or 2 years	0.257	0.013	0.231	0.284
Times smoked in-house	0.118	0.005	0.109	0.127
Times smoked inside enclosed public places	0.151	0.005	0.141	0.160
Times smoked at outdoor public places	−0.007	0.005	−0.015	0.002
Favors ban of smoking at public places	0.968	0.014	0.941	0.995
Advised to stop smoking	1.059	0.015	1.030	1.087
Owns cigarette brand logo	0.386	0.013	0.361	0.410
Attends cigarette ad events	0.636	0.011	0.614	0.659
Offered free cigarettes	0.031	0.014	0.004	0.058
Taught dangers of smoking before	0.367	0.012	0.343	0.391
Number of cigarettes in life	−0.105	0.003	−0.111	−0.099
Cigarettes sold near home	−0.387	0.012	−0.411	−0.363
Youth groups discourage smoking	0.190	0.013	0.163	0.215
Health workers explained smoking dangers	0.464	0.012	0.440	0.488
Religious groups discourage smoking	−0.098	0.013	−0.123	−0.075
Have not tried to stop smoking	−0.311	0.018	−0.345	−0.276
Tried to stop to improve health	0.978	0.016	0.947	1.010
Tried to stop to save money	0.594	0.025	0.546	0.642
Tried to stop because family dislikes it	0.699	0.016	0.669	0.730
Closest friends smoke tobacco	−0.176	0.012	−0.199	−0.153
Both parents smoke	−0.016	0.022	−0.058	0.027
Only father smokes	0.281	0.015	0.251	0.310
Only mother smokes	−1.055	0.028	−1.112	−1.001
Peter Stuyvesant	−0.846	0.024	−0.894	−0.799
Roth man	0.341	0.029	0.283	0.398
Consulate	0.000	0.025	−0.049	0.050
Safari	0.320	0.070	0.183	0.450
From own burns	1.248	0.036	1.178	1.318
Group-level effects	0.658	0.749	0.139	2.774
